# Associations Between Hourly Ambient Particulate Matter Air Pollution and Ambulance Emergency Calls: Time-Stratified Case-Crossover Study

**DOI:** 10.2196/47022

**Published:** 2023-06-20

**Authors:** Qiang Zhou, Hanxu Shi, Rengyu Wu, Hong Zhu, Chongzhen Qin, Zhisheng Liang, Shengzhi Sun, Junfeng Zhao, Yasha Wang, Jie Huang, Yinzi Jin, Zhijie Zheng, Jingyan Li, Zhenyu Zhang

**Affiliations:** 1 Shenzhen Center for Prehospital Care Shenzhen China; 2 Department of Global Health Peking University School of Public Health Beijing China; 3 School of Public Health Capital Medical University Beijing China; 4 School of Computer Science Peking University Beijing China; 5 National Engineering Research Center of Software Engineering Peking University Beijing China; 6 School of Public Health and Emergency Management Southern University of Science and Technology Shenzhen China; 7 Institute for Global Health and Development Peking University Beijing China; 8 China National Environmental Monitoring Centre Beijing China

**Keywords:** particulate matter air pollution, ambulance emergency calls, AECs, environmental epidemiology, public health, air pollution, environmental data, patient data

## Abstract

**Background:**

Associations between short-term exposure to ambient particulate matter (PM) air pollutants and mortality or hospital admissions have been well-documented in previous studies. Less is known about the associations of hourly exposure to PM air pollutants with ambulance emergency calls (AECs) for all causes and specific causes by conducting a case-crossover study. In addition, different patterns of AECs may be attributed to different seasons and daytime or nighttime periods.

**Objective:**

In this study, we quantified the risk of all-cause and cause-specific AECs associated with hourly PM air pollutants between January 1, 2013, and December 31, 2019, in Shenzhen, China. We also examined whether the observed associations of PM air pollutants with AECs for all causes differed across strata defined by sex, age, season, and the time of day.

**Methods:**

We used ambulance emergency dispatch data and environmental data between January 1, 2013, and December 31, 2019, from the Shenzhen Ambulance Emergency Centre and the National Environmental Monitor Station to conduct a time-stratified case-crossover study to estimate the associations of air pollutants (ie, PM with an aerodynamic diameter less than 2.5 µm [PM_2.5_] or 10 µm [PM_10_]) with all-cause and cause-specific AECs. We generated a well-established, distributed lag nonlinear model for nonlinear concentration response and nonlinear lag-response functions. We used conditional logistic regression to estimate odds ratios with 95% CIs, adjusted for public holidays, season, the time of day, the day of the week, hourly temperature, and hourly humidity, to examine the association of all-cause and cause-specific AECs with hourly air pollutant concentrations.

**Results:**

A total of 3,022,164 patients were identified during the study period in Shenzhen. Each IQR increase in PM_2.5_ (24.0 µg/m^3^) and PM_10_ (34.0 µg/m^3^) concentrations over 24 hours was associated with an increased risk of AECs (PM_2.5_: all-cause, 1.8%, 95% CI 0.8%-2.4%; PM_10_: all-cause, 2.0%, 95% CI 1.1%-2.9%). We observed a stronger association of all-cause AECs with PM_2.5_ and PM_10_ in the daytime than in the nighttime (PM_2.5_: daytime, 1.7%, 95% CI 0.5%-3.0%; nighttime, 1.4%, 95% CI 0.3%-2.6%; PM_10_: daytime, 2.1%, 95% CI 0.9%-3.4%; nighttime, 1.7%, 95% CI 0.6%-2.8%) and in the older group than in the younger group (PM_2.5_: 18-64 years, 1.4%, 95% CI 0.6%-2.1%; ≥65 years, 1.6%, 95% CI 0.6%-2.6%; PM_10_: 18-64 years, 1.8%, 95% CI 0.9%-2.6%; ≥65 years, 2.0%, 95% CI 1.1%-3.0%).

**Conclusions:**

The risk of all-cause AECs increased consistently with increasing concentrations of PM air pollutants, showing a nearly linear relationship with no apparent thresholds. PM air pollution increase was associated with a higher risk of all-cause AECs and cardiovascular diseases–, respiratory diseases–, and reproductive illnesses–related AECs. The results of this study may be valuable to air pollution attributable to the distribution of emergency resources and consistent air pollution control.

## Introduction

It is well-known that exposure to ambient particulate matter (PM) causes huge public health burdens due to substantial excess morbidity and mortality [1]. Over the past decade, a growing number of epidemiological studies have examined associations between short-term exposure to air pollutants and the incidence of cardiovascular disease (ie, acute coronary syndrome and myocardial infarction) [2-9]. China’s government has established several policies to control air pollution with great effort, and the severity of air pollution has been eliminated to some extent. However, with continued urbanization and climate change, air pollution still requires focused efforts [10,11]. Therefore, the burden of disease associated with air pollutants is substantial and should be the focus of further research.

Although previous studies have documented the effects of short-term daily air pollution exposure on cardiovascular diseases, less is known about the effects of hourly air pollution exposure using a case-crossover study design. Moreover, most comprehensive studies on air pollutants’ health effects have focused on cardiovascular or respiratory diseases–related mortality or hospital admissions, with limited evidence on assessing all-cause health impacts on ambulance emergency calls (AECs). Di et al [12] conducted a study that included 22,433,862 case-days and 76,143,209 control-days and found that each short-term increase of 10 μg/m^3^ was statistically significantly associated with a relative increase of 1.05% (95% CI 0.95%-1.15%) in the daily mortality rate from 2000 to 2012. Ito et al [13] conducted a time-series study that observed a positive association between PM with an aerodynamic diameter less than 2.5 µm [PM_2.5_], daily deaths, and emergency hospitalizations for cardiovascular diseases in New York City. AECs may serve as a more sensitive indicator of the health effects of air pollutants and a more appropriate signal for syndromic surveillance [14,15]. In addition, different patterns of AECs may be attributed to different seasons and daytime or nighttime periods. Identifying these potential patterns can be useful in helping people avoid exposure, but more evidence is needed. Although a few previous studies investigated hourly PM air pollutants’ association with disease onset, limitations such as a small sample size with a short period, the lack of all-cause disease as a variable, and only focusing on PM_2.5_ should not be ignored. For example, one Canadian study that only included 500,302 individuals found that exposure to elevated hourly PM_2.5_ during wildfire seasons was associated with increased odds of dispatches related to respiratory and cardiovascular conditions over 5 years [16]. Therefore, it is imperative to conduct a time-stratified case-crossover study that includes a relatively large sample size with a long study period to investigate the associations of PM air pollutants with all-cause and cause-specific AECs. In this study, we quantified the risk of all-cause and cause-specific AECs associated with hourly air pollutants (ie, PM_2.5_ and PM with an aerodynamic diameter less than 10 µm [PM_10_]) between January 1, 2013, and December 31, 2019, in Shenzhen, China. We also examined whether the observed associations of PM air pollutants with AECs for all causes differed across strata defined by sex, age, season, and the time of day.

## Methods

### Study Population

Shenzhen is a major city located in the Pearl River Delta region of China, adjacent to Hong Kong. As one of the country’s 4 province-level municipalities, Shenzhen has a population of 17.68 million and a gross domestic product per capita of CNY ¥173,700 (US $24,446) as of 2021. The Shenzhen Center for Prehospital Care developed the 120 Emergency Medical Services system in 1994, which includes 73 emergency networks and 103 emergency stations. In 2022, the center received 2,278,232 all-cause emergency calls, an increase of 41.96% from the previous year, and had 295,295 emergency dispatches, an increase of 14.23% from the previous year. Shenzhen has a subtropical monsoon climate that is typical of southern areas of China. We recruited individuals aged >18 years who called the emergency ambulance to reach the hospital, and those with time records linked to transfer were eligible to be included during the period from 2013 to 2019. We used ambulance emergency dispatch data and environmental data between January 1, 2013, and December 31, 2019, from the Shenzhen Ambulance Emergency Centre and the National Environmental Monitor Station, which included patients’ self-reported medical claims, the time of AECs, prehospital diagnosis, demographic characteristics, and hourly air pollutant concentrations. Each call in the emergency data set was linked to a patient care report that was completed by the attending paramedics.

### Study Design

We used a time-stratified case-crossover study design to estimate the associations of PM pollutants (ie, PM_2.5_ and PM_10_) with all-cause and cause-specific AECs. Each patient was exposed to PM pollutants before the hour in which the AEC occurred, compared to control periods of the same individual when the AEC did not occur [17,18]. This study design allows for the elimination of potential confounding from all known and unknown time-invariant factors (ie, age, sex, ethnicity, behavioral factors, and socioeconomic status) and covariant factors that vary slowly, such as seasonality [19]. We determined that each case index hour was the hour in which the AEC occurred (ie, case period), and we used 3 or 4 control index hours to match this hour by the same hour of the day, day of the week, month, and year with the case index hour to control for long-term time trends [12]. For example, if the first AEC occurred at 6 AM on Monday, May 20, 2013, we would define 6 AM on Monday, May 20, 2013, as the case index hour and 6 AM on all other Mondays in May 2013 (May 6, 13, and 27) as the control index hours.

### Environmental Data Ascertainment

We obtained hourly concentrations of air pollutants (ie, PM_2.5_ and PM_10_) from the National Environmental Monitor Station during the study period. The station provides real-time data on criteria air pollutants across all nationally controlled monitoring stations operated by the China National Environmental Monitoring Centre with strict standard data quality control procedures. To facilitate communication, we first defined each IQR increase as the difference between the 25th and 75th percentile of air pollutant concentrations [20]. We also defined the extreme concentration (ie, PM_2.5_ and PM_10_) as the maximum concentration equal to the 97.5th percentile of concentrations over the study period. We calculated a population-weighted average of each IQR concentration increase and the maximum hourly concentration for each hour [21].

### AECs Ascertainment

We combined patients’ self-reported medical claims and prehospital diagnosis as the final diagnosis. Our primary outcome was all-cause AECs, which we further classified into 3 cause-specific categories: AECs related to cardiovascular diseases, respiratory diseases, and reproductive illnesses. For instance, we classified hypertension, acute ischemic stroke, myocardial infarction, ischemic heart disease, acute coronary syndrome, and unstable angina as cardiovascular diseases–related AECs, whereas calls due to lung diseases, bronchitis, respiratory difficulties, and asthma were categorized as respiratory diseases–related AECs. Similarly, calls related to pregnancy, abortion, or other reproductive disorders were classified as reproductive illnesses–related AECs. Additionally, we created a separate category for AECs due to injury, poisoning, mental health disorders, endocrine disorders, and digestive disorders. We were careful to exclude any vague claims such as dizziness, abdominal pain, discomfort, unconsciousness, headache, and allergy while generating disease stratification. We excluded patients who had both missing self-reported claims and missing prehospital diagnoses. We extracted the relevant demographic information (ie, age, sex, and year) and admission date for each diagnosis. We calculated cause-specific AECs by 24 hours and all-cause AECs by age to reflect the hourly distribution of AECs.

### Statistical Analysis

We generated a well-established, distributed lag nonlinear model for nonlinear concentration-response and nonlinear lag-response functions [16]. We modeled the concentration-response function using a natural cubic B-spline with 1 or 2 knots to account for potential nonlinear relationships. We also modeled the lag-response function using a linear function with 1 or 2 knots placed on the log scale of lags up to 48 hours. We used conditional logistic regression to estimate odds ratios (ORs) with 95% CIs, examining the association of all-cause and cause-specific AECs with hourly air pollution concentration. The following covariates were adjusted in our main models: natural spline functions with 3 degrees of freedom for temperature and humidity, public holidays, and the day of the week. We converted ORs with 95% CIs to percentage changes in the risk of all-cause and cause-specific AECs associated with each IQR increase in air pollutant concentrations [20,22,23]. The following equations were used:

Percentage change IQR = (e^β × IQR^ – 1) × 100%
**(1)**

Lower 95% CI = (e^[β – 1.96 × SE] × IQR^ – 1) × 100%
**(2)**

Upper 95% CI = (e^[β + 1.96× SE] × IQR^ – 1) × 100%
**(3)
**

where β is the regression coefficient.

We conducted several stratified analyses by age (18-64 vs ≥65 years), sex (male vs female), season (warm vs cool), and the time of day (daytime vs nighttime) to examine potential effect modifications. The warm season ranged from March to October and the cool season ranged from November to February in Shenzhen. Nighttime is defined as being from 8:00 PM to 7:00 AM in the next day, and daytime is defined as being from 8:00 AM to 7:00 PM within one day [20]. Missing rates of PM air pollutants were less than 0.51%, and we filled in missing data by using the next or previous entry.

We conducted a number of sensitivity analyses to assess the robustness of our results. First, we examined the correlations between several air pollutants to estimate the multicollinearity. Second, we repeated the main analyses based on exposure to the extreme concentration, rather than using only each IQR increase, to observe the robustness of the exposure metric. Third, we used variable key modeling parameters, including modelling the concentration-response functions using a natural B-spline with 3 internal knots for all-cause AECs and 2 or 3 knots for cause-specific AECs and the lag-response function using a natural cubic B-spline with 4 knots for all-cause AECs and 2 or 3 knots for cause-specific AECs placed on the log scale of lags up to 48 hours.

All analyses were performed in R (version 4.2.1; R Foundation for Statistical Computing). The *survival* package was used for conditional logistic regression, and the *dlnm* package was used for the distributed lag nonlinear model.

### Ethics Approval

Ethics approval and consent to participate in this project was approved by the Peking University Health Science Center Institutional Review Board (PUIRB-YS2023123). Informed consent was obtained from all participants prior to questionnaire administration.

## Results

**Figure 1:**
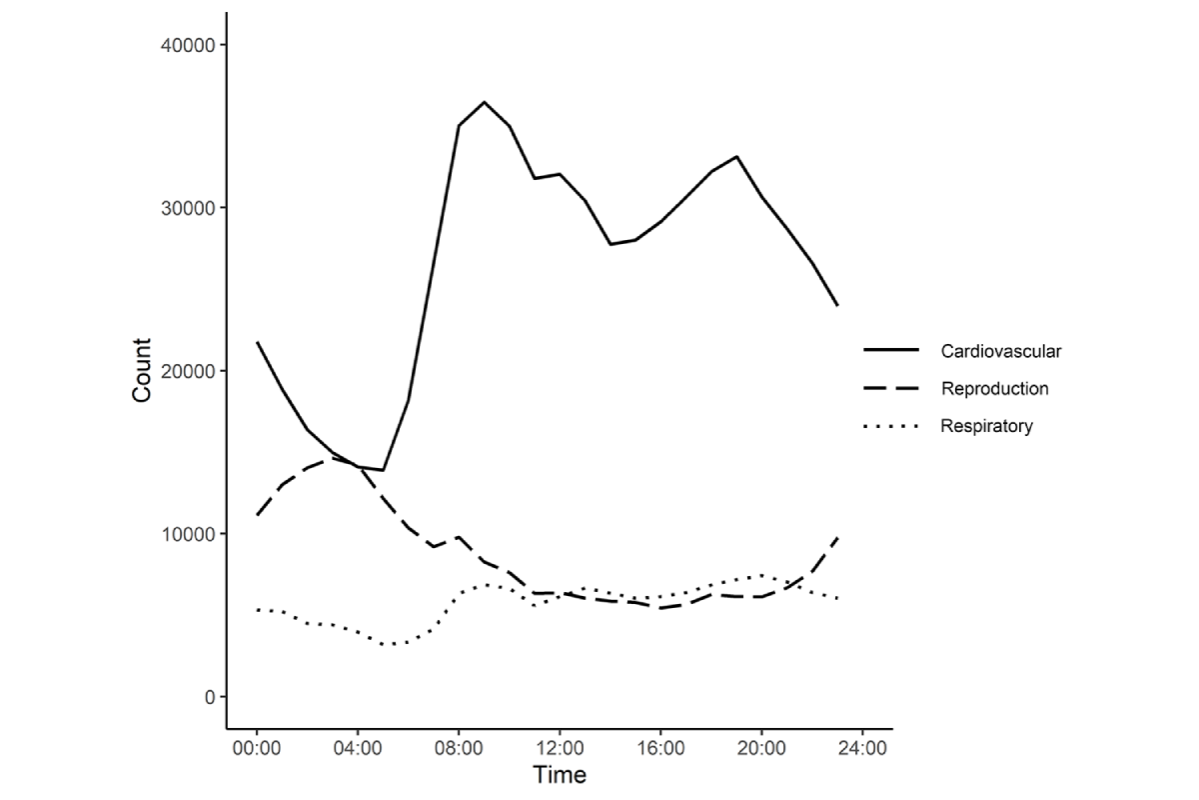
The distribution of different types of ambulance emergency calls among 3,022,164 patients over 24 hours in Shenzhen from 2013 to 2019.

A total of 3,022,164 patients were identified during the study period in Shenzhen: 64.3% (n=1,942,832) were male, 81.5% (n=2,462,968) were aged 20-64 years, 21.1% (n=636,288) made AECs due to cardiovascular diseases, and 51.4% (n=1,553,480) called the ambulance in the daytime (Table 1). The average hourly concentrations of PM_2.5_ (32.2 µg/m^3^) and PM_10_ (49.6 µg/m^3^) before the index hour were well above the recently updated World Health Organization Global Air Quality Guidelines 2021 [24] (annual average: PM_2.5_, 5.0 µg/m^3^; PM_10_, 10.0 µg/m^3^; Table S1 in Multimedia Appendix 1). AECs due to cardiovascular diseases rode 2 crests at 9:00 AM and 8:00 PM over 24 hours, the fluctuations of AECs due to respiratory diseases were relatively gentle, and AECs due to reproductive illnesses peaked at 3:00 AM and 11:00 PM (Figure 1). We found significant differences in all-cause and cause-specific AECs between daytime and nighttime, stratified by age (Table 2). For example, the proportion of all-cause AECs was 34.5% (n=128,928) in the ≥65 years group and 49% (n=90,792; <20 years) and 54.1% (n=1,333,684; 20-64 years) in the younger groups at nighttime. We found that all-cause AECs reached their zeniths at 7:00 AM and 9:00 AM in the ≥65 years group, and the 20-64 years group reached their peaks at 6:00 AM and 10:00 PM over 24 hours (Figure S1 in Multimedia Appendix 1).

**Table 1:** Baseline characteristics of populations in Shenzhen from 2013 to 2019.

Baseline characteristics			Patient (N=3,022,164), n (%)
**Age (years)**			
	<20	185,152 (6.1)	
	20-64	2,462,968 (81.5)	
	≥65	373,884 (12.4)	
**Sex**			
	Male	1,942,832 (64.3)	
	Female	1,079,256 (35.7)	
**Cases by year**			
	2013	355,816 (11.8)	
	2014	385,232 (12.7)	
	2015	399,756 (13.2)	
	2016	429,948 (14.2)	
	2017	465,060 (15.4)	
	2018	475,104 (15.7)	
	2019	511,248 (17)	
**Cases by types of AECs^a^**			
	Cardiovascular	636,288 (21.1)	
	Respiratory	137,960 (4.6)	
	Reproductive	208,408 (6.9)	
	Others^b^	2,039,508 (67.4)	
**Time of day^c^**			
	Daytime	1,553,480 (51.4)	
	Nighttime	1,468,684 (48.6)	

^a^AEC: ambulance emergency call.

^b^Others: ambulance emergency calls due to injury, poisoning, mental health disorders, endocrine disorders, and digestive disorders.

^c^Time of day: daytime is from 8:00 AM to 7:00 PM within one day; nighttime is from 8:00 PM to 7:00 AM in the next day.

**Table 2:** Percentage of all types of ambulance emergency calls (AECs) by the time of day and age group in Shenzhen from 2013 to 2019 (N=3,022,164).

AECs		Daytime^a^, n (%)	Nighttime^a^, n (%)	*P* value
**Total population**				
	All-cause	1,468,684 (48.6)	1,553,480 (51.4)	<.001
	Cardiovascular	381,668 (60)	254,620 (40)	<.001
	Respiratory	77,072 (55.9)	60,868 (44.1)	<.001
	Reproductive	79,484 (38.1)	128,924 (61.9)	<.001
**<20 years group**				
	All-cause	94,360 (51)	90,792 (49)	<.001
	Cardiovascular	22,208 (62.7)	13,204 (37.3)	<.001
	Respiratory	1,0916 (55)	8920 (45)	<.001
	Reproductive	3620 (44.3)	4552 (55.7)	<.001
**20-64 years group**				
	All-cause	1,129,284 (45.9)	1,333,684 (54.1)	<.001
	Cardiovascular	257,016 (58.8)	180,048 (41.2)	<.001
	Respiratory	35,624 (52.2)	32,628 (47.8)	<.001
	Reproductive	72,188 (37.2)	121,716 (62.8)	<.001
≥**65 years group**				
	All-cause	244,956 (65.5)	128,928 (34.5)	<.001
	Cardiovascular	102,436 (62.5)	61,360 (37.5)	<.001
	Respiratory	30,532 (61.2)	19,320 (38.8)	<.001
	Reproductive	—^b^	—	—

^a^Daytime is from 8:00 AM to 7:00 PM within one day; nighttime is from 8:00 PM to 7:00 AM in the next day.

^b^Not applicable.

There were moderate to high correlations among air pollutants (Table S2 in Multimedia Appendix 1). PM_2.5_ was positively correlated with PM_10_, nitrogen dioxide, ozone, and sulfur dioxide (Spearman *r*=0.93, 0.61, 0.56, and 0.62, respectively), and PM_10_ was positively correlated with PM_2.5_, nitrogen dioxide, ozone, and sulfur dioxide (Spearman *r*=0.93, 0.64, 0.52, and 0.66, respectively).

**Figure 2:**
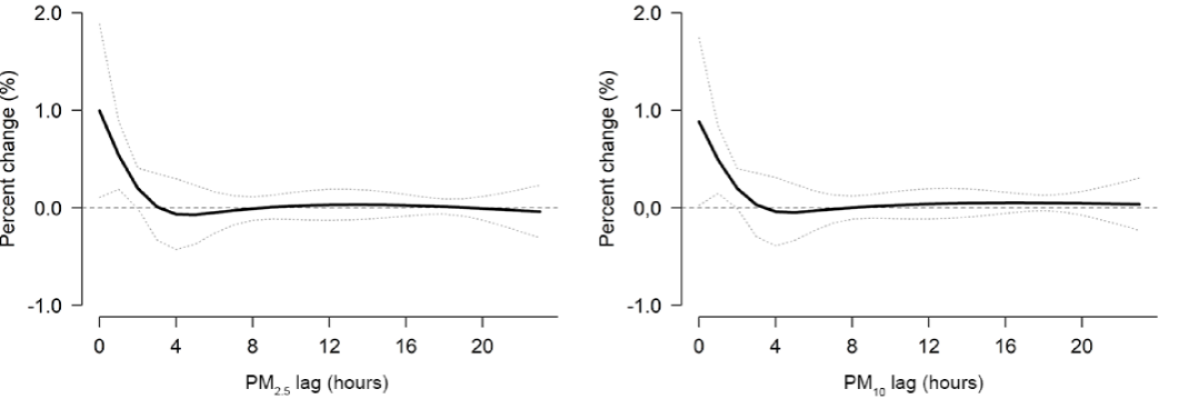
Lag structures for the associations of all-cause ambulance emergency calls with each IQR increase in PM_2.5_ and PM_10_ concentration over lags of up to 24 hours in Shenzhen from 2013 to 2019. The overall lag structure curves are calculated using a linear with 2 knots placed on the log scale of lags to model the lag-response association. The solid black lines are the average percentage change in the risk of all-cause ambulance emergency calls with each IQR increase in PM_2.5_ and PM_10_ concentration (24.0 µg/m^3^ and 34.0 µg/m^3^, respectively), and the dotted lines are the 95% CIs. PM_2.5_ and PM_10_: particulate matter less than 2.5 and 10 µm in diameter, respectively.

**Figure 3:**
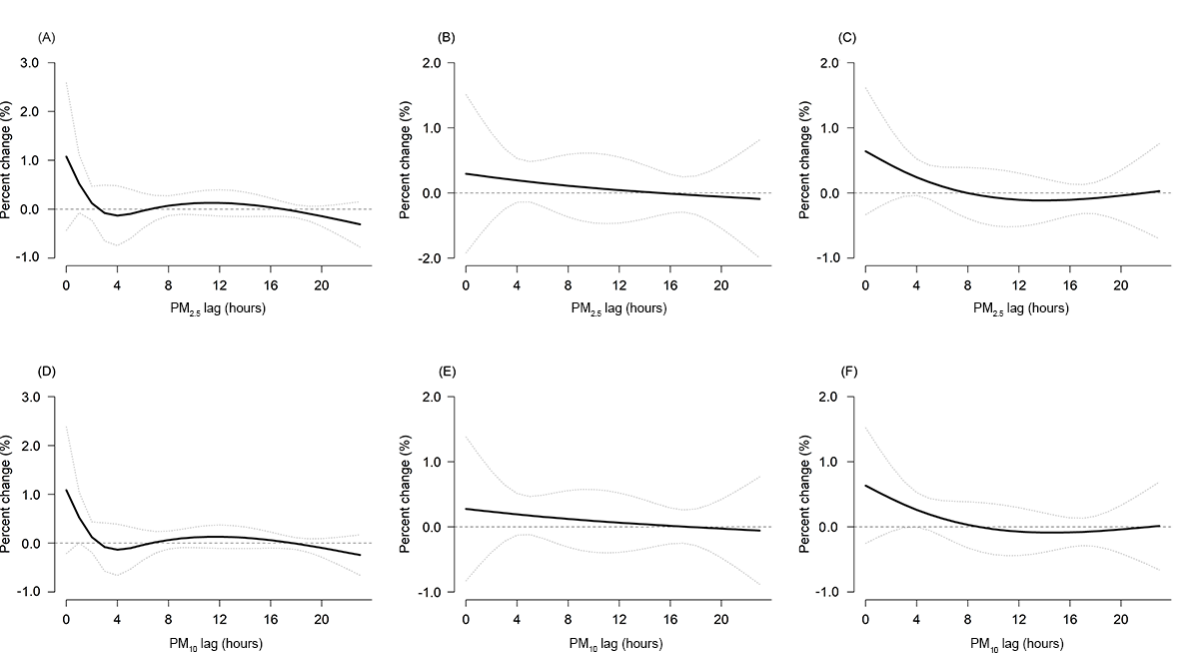
Lag structures for the associations of ambulance emergency calls due to cardiovascular, respiratory, and reproductive diseases with each IQR increase in PM_2.5_ and PM_10_ concentration over lags of up to 24 hours in Shenzhen from 2013 to 2019. A and D represent cardiovascular diseases, B and E represent respiratory diseases, and C and F represent reproductive illnesses. The overall lag structure curves are calculated using a linear with 1 or 2 knots placed on the log scale of lags to model the lag-response association. The black solid lines are the average percentage change in the risk of ambulance emergency calls due to cardiovascular, respiratory, and reproductive diseases with each IQR increase in PM_2.5_ and PM_10_ concentration (24.0 µg/m^3^ and 34.0 µg/m^3^, respectively), and the dotted lines are the 95% CIs. PM_2.5_ and PM_10_: particulate matter less than 2.5 and 10 µm in diameter, respectively.

We found associations of exposure to PM_2.5_ and PM_10_ with a higher incidence of all-cause AECs in the concurrent hour. After that, the associations gradually eliminated and became not statistically significant after approximately 15 to 48 hours (Table 3), so we decided to use the duration of 0 to 24 hours to derive the risk estimates (Figures 2 and 3). Each IQR increase in the concentrations of PM_2.5_ (24.0 µg/m^3^) and PM_10_ (34.0 µg/m^3^) in 24 hours was associated with a higher risk of AECs (PM_2.5_: all-cause, 1.8%, 95% CI 0.8%-2.4%; cardiovascular diseases, 1.9%, 95% CI 0.2%-3.1%; respiratory diseases, 2.5%, 95%CI –0.9% to 6.1%; reproductive illnesses, 1.9%, 95% CI 1.0%-4.8%; PM_10_: all-cause, 2.0%, 95% CI 1.1%-2.9%; cardiovascular diseases, 2.0%, 95% CI 0.6%-3.4%; respiratory illnesses, 2.9%, 95% CI –0.6% to 6.6%; reproductive illnesses, 2.1%, 95% CI 0.8%-5.1%; Table 3 and Figures 2 and 3).

**Figure 4:**
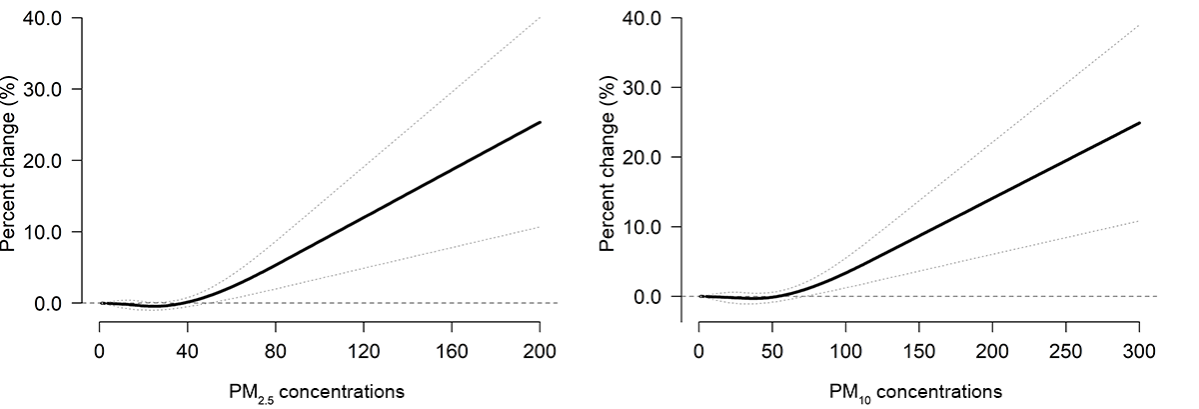
Cumulative concentration-response curves for the association of all-cause ambulance emergency calls with PM_2.5_ and PM_10_ over lags of up to 24 hours in Shenzhen from 2013 to 2019. The cumulative exposure-response curves are calculated using a natural B-spline with 2 knots to model the exposure-response association. The black solid lines are the average percentage change in the risk of all-cause ambulance emergency calls, and the dotted lines are the 95% CIs. PM_2.5_ and PM_10_: particulate matter less than 2.5 and 10 µm in diameter, respectively.

**Figure 5:**
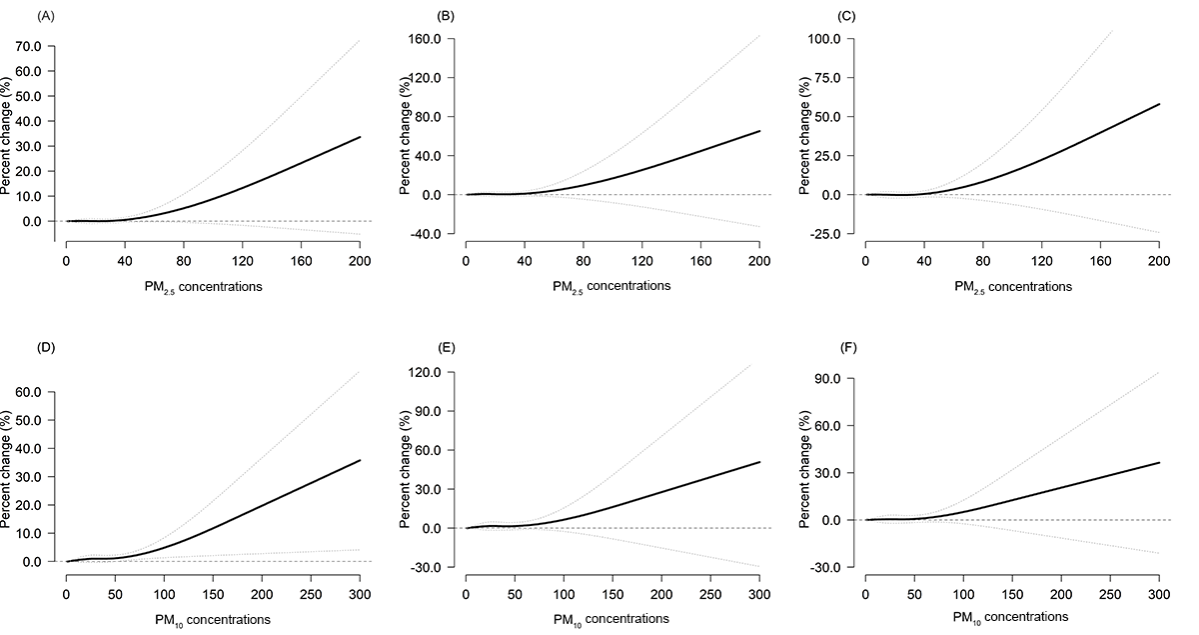
Cumulative concentration-response curves for the associations of ambulance emergency calls due to cardiovascular, respiratory, and reproductive diseases with PM_2.5_ and PM_10_ over lags of up to 24 hours in Shenzhen from 2013 to 2019. A and D represent cardiovascular diseases, B and E represent respiratory diseases, and C and F represent reproductive illnesses. The cumulative exposure-response curves are calculated using a natural B-spline with 1 or 2 knots to model the exposure-response association. The black solid lines are the average percentage change in the risk of ambulance emergency calls due to cardiovascular, respiratory, and reproductive diseases with PM2.5 and PM_10_, and the dotted lines are the 95% CIs. PM_2.5_ and PM_10_: particulate matter less than 2.5 and 10 µm in diameter, respectively.

Figures 4 and 5 show the concentration-response curves of all-cause and cause-specific AECs for PM_2.5_ and PM_10_. The risk of all-cause AECs increased consistently with increasing concentrations of PM air pollutants, showing a nearly linear relationship with no apparent thresholds. In general, the risk of all-cause and cause-specific AECs increased dramatically with the accumulated concentration of PM_2.5_ and PM_10_. The magnitude of the associations varied slightly for the cardiovascular diseases–related AECs, whereas the association had a difference in respiratory diseases– and reproductive illness–related AECs.

In stratified analyses, we observed a stronger association of all-cause AECs with PM_2.5_ and PM_10_ in the daytime than in the nighttime (PM_2.5_: daytime, 1.7%, 95% CI 0.5%-3.0%; nighttime, 1.4%, 95% CI 0.3%-2.6%; PM_10_: daytime, 2.1%, 95% CI 0.9%-3.4%; nighttime, 1.7%, 95% CI 0.6%-2.8%) and in the older group than the younger group (PM_2.5_: 18-64 years, 1.4%, 95% CI 0.6%-2.1%; ≥65 years, 1.6%, 95% CI 0.6%-2.6%; PM_10_: 18-64 years, 1.8%, 95% CI 0.9%-2.6%; ≥65 years, 2.0%, 95% CI 1.1%-3.0%; Table 4). For subtypes of AECs, we found stronger associations of respiratory diseases– and reproductive illnesses–related AECs with PM_2.5_ and PM_10_ in the daytime than in the nighttime and weaker associations of cardiovascular diseases–related AECs with PM_2.5_ and PM_10_ in the daytime than in the nighttime (Table 4). Cardiovascular diseases–related AECs with PM_2.5_ and PM_10_ had stronger association in the older group than in the younger group (Table 4).

A series of sensitivity analyses showed that the results were consistent with each IQR increase when the extreme concentration was considered as the exposure metric (Tables S3 and S4 and Figures S2-S7 in Multimedia Appendix 1). The results were consistent when we adopted an alternative number of knots or degrees of freedom for the distribution of air pollution concentration.

**Table 3:** Risk of ambulance emergency calls (AECs) associated with each IQR^a^ increase in PM2.5^b^ and PM10^c^ concentration over different lags in Shenzhen from 2013 to 2019. The models were adjusted for public holidays, days of the week, hourly temperature, and hourly humidity.

AECs	0-12 hours, percentage change (95% CI)			0-24 hours, percentage change (95% CI)			0-36 hours, percentage change (95% CI)			0-48 hours, percentage change (95% CI)	
	PM_2.5_	PM_10_	PM_2.5_		PM_10_	PM_2.5_		PM_10_	PM_2.5_		PM_10_
All-cause	1.7 (0.9 to 2.6)	1.8 (1.0 to 2.6)	1.8 (0.8 to 2.4)		2.0 (1.1 to 2.9)	1.4 (0.5 to 2.4)		1.8 (0.9 to 2.8)	1.3 (0.5 to 2.2)		1.7 (0.9 to 2.6)
Cardiovascular	1.8 (0.3 to 3.2)	1.8 (0.4 to 3.2)	1.9 (0.2 to 3.1)		2.0 (0.6 to 3.4)	1.4 (–0.2 to 3.1)		1.9 (0.3 to 3.6)	1.4 (–0.1 to 2.9)		1.8 (0.3 to 3.3)
Respiratory	2.0 (–1.4 to 5.5)	2.0 (–1.4 to 5.5)	2.5 (–0.9 to 6.1)		2.9 (–0.6 to 6.6)	2.4 (–1.3 to 6.3)		2.2 (–0.6 to 6.7)	1.1 (–1.5 to 5.9)		1.8 (–0.9 to 6.7)
Reproductive	2.3 (0.3 to 5.3)	2.3 (0.1 to 5.3)	1.9 (1.0 to 4.8)		2.1 (0.8 to 5.1)	1.4 (–1.6 to 4.6)		1.9 (–1.2 to 5.1)	2.5 (–0.5 to 5.5)		2.8 (–0.2 to 5.9)

^a^IQR: IQR of PM_2.5_ was defined as the 25th to 75th percentile (24.0 µg/m^3^); IQR of PM_10_ was defined as the 25th to 75th percentile (34.0 µg/m^3^).

^b^PM_2.5_: particulate matter less than 2.5 µm in diameter.

^c^PM_10_: particulate matter less than 10 µm in diameter.

**Table 4:** Risk of ambulance emergency calls associated with each IQR^a^ increase in PM2.5^b^ and PM10^c^ concentrations over lags of 0-24 hours, stratified by sex, age, season, and the time of day, in Shenzhen from 2013 to 2019. The models were adjusted public holidays, days of the week, hourly temperature, and hourly humidity.

Subgroups		All-cause, percentage change (95% CI)			Cardiovascular diseases, percentage change (95% CI)			Respiratory diseases, percentage change (95% CI)			Reproductive illnesses, percentage change (95% CI)	
		PM_2.5_	PM_10_	PM_2.5_		PM_10_	PM_2.5_		PM_10_	PM_2.5_		PM_10_
**Sex**												
	Male	1.5 (0.7 to 2.3)	1.8 (0.9 to 2.6)	1.6 (0.1 to 3.0)		1.9 (0.5 to 3.4)	2.5 (–1.2 to 6.4)		2.8 (–0.8 to 6.5)	2.0 (–0.8 to 4.9)		2.3 (–0.6 to 5.2)
	Female	1.5 (0.7 to 2.4)	1.8 (1.0 to 2.7)	1.6 (0.2 to 3.1)		1.9 (0.5 to 3.4)	2.6 (–1.2 to 6.5)		2.8 (–0.8 to 6.5)	2.1 (–0.7 to 5.0)		2.3 (–0.6 to 5.3)
**Age group (years)**												
	18-64	1.4 (0.6 to 2.1)	1.8 (0.9 to 2.6)	1.4 (0.1 to 2.8)		1.9 (0.5 to 3.4)	2.3 (–1.2 to 5.9)		2.7 (–0.8 to 6.4)	2.0 (–0.8 to 4.9)		3.7 (–2.1 to 9.9)
	≥65	1.6 (0.6 to 2.6)	2.0 (1.1 to 3.0)	1.7 (0.2 to 3.3)		2.2 (0.6 to 3.8)	2.4 (–1.6 to 6.6)		2.8 (–1.1 to 6.8)	1.9 (–1.5 to 5.6)		2.5 (–1.2 to 6.3)
**Season^d^**												
	Warm	1.5 (0.7 to 2.3)	1.8 (0.9 to 2.7)	0.6 (0.2 to 2.8)		1.2 (–0.4 to 2.8)	2.6 (–0.9 to 6.2)		2.8 (–1.3 to 7.0)	3.6 (–2.3 to 9.8)		3.7 (–2.6 to 10.4)
	Cool	1.4 (–2.7 to 3.6)	–6.4 (–21.2 to 6.4)	1.6 (–0.2 to 3.4)		–12.0 (–34.0 to 9.4)	1.3 (–2.6 to 5.4)		1.6 (–3.3 to 6.7)	1.0 (–2.8 to 5.1)		2.6 (–6.5 to 12.5)
**Time of day^e^**												
	Daytime	1.7 (0.5 to 3.0)	2.1 (0.9 to 3.4)	1.4 (–0.1 to 3.2)		1.5 (0.2 to 3.9)	2.7 (–2.1 to 7.7)		3.1 (–1.4 to 7.9)	1.5 (–3.0 to 6.3)		2.2 (–2.7 to 7.3)
	Nighttime	1.4 (0.3 to 2.6)	1.7 (0.6 to 2.8)	1.6 (–1.7 to 3.8)		1.9 (–0.4 to 4.2)	1.6 (–3.0 to 8.4)		2.9 (–3.0 to 9.2)	5.7 (–2.9 to 15.2)		5.3 (–3.4 to 14.7)

^a^IQR: IQR of PM_2.5_ was defined as the 25th to 75th percentile (24.0 µg/m^3^); IQR of PM_10_ was defined as the 25th to 75th percentile (34.0 µg/m^3^).

^b^PM_2.5_: particulate matter less than 2.5 µm in diameter.

^c^PM_10_: particulate matter less than 10 µm in diameter.

^d^Season: warm season is from 1^st^ April 1 to October 31 of each year; cool season is from November 1 to 31 March 31 of each year.

^e^Time of the day: daytime is from 8:00 AM to 7:00 PM within one day; nighttime is from 8:00 PM to 7:00 AM in the next day.

## Discussion

### Principal Findings

We observed the associations of transient exposure to air pollutants with an increased risk of all-cause AECs, without any discernible threshold effects. The associations were stronger for PM_2.5_ than for PM_10_. In addition, the risks were apparent and statistically significant within 0-4 hours after exposure to the air pollutants. The excess relative risks showed substantial differences between the time-of-day strata.

Our findings were consistent with a robust body of previous studies demonstrating the positive associations of hourly air pollutants with all-cause and cause-specific AECs [15,16,23,25-30]. However, much of the existing evidence has focused on assessing the associations between cardiovascular diseases–related hospital admissions and exposures at the daily timescale through time-series analyses [31,32]. We have filled these knowledge gaps and generalized these results to the emergency medical service setting, as this study demonstrated that AECs may serve as a sensitive and timely marker of the adverse health effects of air pollution.

Given the use of different exposure metrics, time periods, study designs, and analytical strategies, our results cannot be compared directly across studies. For example, an analysis of Shenzhen AECs between January 18, 2013, and December 31, 2016, found that each 10 µg/m^3^ increase in PM_2.5_ and PM_10_ was associated with a 1.44% (95% CI, 0.70-2.19%) and 0.95% (95% CI, 0.39-1.51%) increase in hourly AECs over 5 hours, respectively [23]. In another study in Shenzhen [25], the authors observed positive associations of exposure to PM_2.5_ with an increased risk of emergency department visits across different lag days using daily excessive concentration hours and the daily mean metric. In contrast, relatively weak effects were found for hourly peak PM_2.5_. Rao et al [33] conducted a case-crossover study by using British Columbia Emergency Health Service data from 2010 to 2015 in Canada and showed a positive association between cardiovascular disease–related AECs and PM_2.5_ exposure (OR 1.007, 95% CI 0.997-1.031), and there was a slight increase in the odds of respiratory disease–related AECs at 1.005 (95% CI 0.998, 1.013) over 48 hours. Another study conducted by Ai et al [15] between 2014 and 2016 found that each 10 µg/m^3^ increase in PM_2.5_ and PM1_0_ was associated with a 0.19% (95% CI 0.03-0.35%) and 0.13% (95% CI, 0.02-0.24%) increase of all-cause AECs, respectively, whereas no significant effects of PM_2.5_ and PM_10_ on cardiovascular morbidity were found.

We observed significant effects during the first 24 hours after exposure to the PM pollutants. Several human and animal studies have shown that acute exposure to PM may enhance thrombogenicity through various pathways, including platelet activation, oxidative stress, and the interplay between interleukin-6 and tissue factors [30,34,35]. A few studies have also identified the possible pathways, including endothelial dysfunction, inflammation, dyslipidemia, and autonomic and vascular dysfunction [33,36].

The stratified analyses showed stronger associations of air pollutants with cardiovascular diseases–related AECs in the nighttime than in the daytime, which is in line with existing evidence [22,37]. This may be due to low atmospheric pressure that always occurs during the night (from 1:00 AM to 5:00 AM). Moreover, we found several significant season differences in associations of PM air pollutants with respiratory diseases–related AECs, which could be explained by the virus or allergen being more active in the warm season. Our analysis observed stronger associations of all-cause AECs in the daytime, this could be explained by people often spending more time outdoors during the day and that the associations were estimated based on fixed-site monitors [20]. In addition, there were no associations of air pollutants with AECs stratified by sex in our study. These results were in line with previous literature [7,17], indicating that all patients, regardless of sex, seem to be at higher AEC risks after transient exposure to air pollution.

This study has several limitations. First, we used the individual weighted average of hourly air pollution concentrations as a proxy for personal exposure. Although there is some degree of exposure misclassification that may lead to the underestimation of associations, this limitation may not be avoidable in most time-series studies [22,38-40]. Second, we used prehospital diagnosis rather than medical diagnosis with specific International Classification of Disease codes because data on the final clinical diagnosis were not available. However, we defined our subtypes of AECs using the Medical Priority Dispatch System [16,41], which should not substantially bias our results. Third, we did not have detailed information on the locations of environmental monitoring stations and the proximity of roads, making it challenging to explore the possible effect modification of the associations. Fourth, we were unable to match the monitor station measurements to the closest patient because we were unable to acquire patients’ location information. Therefore, we used the average city-level PM concentration as the exposure for patients in this study. Although the impact of this limitation is likely minimal, we plan to gather and analyze this association in future studies [20].

This study has some strengths. First, the Shenzhen Ambulance Emergency Centre provided high-quality data covering most hospitals in Shenzhen. The large sample size and individual-level data allowed us to conduct comprehensive statistical analyses to maximize the validity of the study results. Second, the time-stratified case-crossover study design facilitated causal inference of the study results by adjusting for time-invariant confounding [18,40]. Third, we collected hourly data on air pollutants when all-cause or cause-specific AECs occurred, which characterized the subdaily time effects of exposure to AECs.

Air pollution–related AECs are considered as a public health problem. This study provides the distributions of all-cause and cause-specific AECs over 24 hours and the associations of each IQR increase with AEC risks. The adverse health effects of air pollution are preventable through a combination of reducing exposure, reducing susceptibility, and improving adaptive capacity. In the context of public health preparedness for air pollution, local government response systems should typically provide air pollution–related information to the public, develop strategies to reduce air pollution–related risks, and allocate emergency ambulance resources equitably. It is also important for hospitals and emergency centers to adapt their procedures to meet the increased demands associated with air pollution. For example, local health systems need not only to allocate more resources (ie, health care providers and medical facilities) to general emergency care but also to increase the capacity of health care providers to provide specialized emergency care (ie, cardiovascular and respiratory diseases) at night because of the stronger associations of PM air pollutants found during nighttime hours.

### Conclusion

PM air pollutants were associated with a higher relative risk of all-cause AECs and cardiovascular diseases–, respiratory diseases–, and reproductive illnesses–related AECs. The risk of all-cause AECs increased consistently with increasing concentrations of PM air pollutants, showing a nearly linear relationship with no apparent thresholds. The results of this study may be valuable to air pollution attributable to the distribution of emergency resources and consistent air pollution control.

## Appendix

Multimedia Appendix 1 Hourly particulate matter concentrations, ambulance emergency call distribution, air pollutant correlations, and sensitivity analyses.

## Abbreviations

AEC: ambulance emergency call
OR: odds ratio
PM: particulate matter
PM10: particulate matter less than 10 µm in diameter
PM2.5: particulate matter less than 2.5 µm in diameter

## Data Availability

The data sets generated and/or analyzed during the current study are not publicly available owing to security protocols and privacy regulations but are available from the corresponding author on reasonable request.
