# Supplementary material for: Associations Between Hourly Ambient Particulate Matter Air Pollution and Ambulance Emergency Calls: Time-Stratified Case-Crossover Study
Source: JMIR Public Health Surveill. 2023 Jun 20;9:e47022. doi: 10.2196/47022 (PMC10337377; doi:10.2196/47022)
Supplement: Multimedia Appendix 1 [file publichealth_v9i1e47022_app1.docx]

**Supplementary Online Content**

**Table S1.** Distributions of hourly PM_2.5_ & PM_10_ over lags 0-24h prior to the index hour in Shenzhen from 2013-19

**Table S2.** Spearman correlation coefficients between air pollutants at an hourly level

**Table S3.** Risk of ambulance emergency calls associated with extreme concentration in PM_2.5_ & PM_10_ over different lags in Shenzhen from 2013-19

**Table S4.** Risk of ambulance emergency calls associated with extreme concentration in PM_2.5_ & PM_10_ over lags 0-24h, stratified by sex, age, season, and time of day in Shenzhen from 2013-19

**Figure S1.** All-cause ambulance emergency calls’ distribution of 3,022,164 patients over 0-24h, stratified by age in Shenzhen from 2013-19

**Figure S2.** Lag structures for the associations of all-cause ambulance emergency calls with extreme concentration in PM_2.5_ & PM_10_ over lags 0-24h in Shenzhen from 2013-19

**Figure S3.** Lag structures for the associations of ambulance emergency calls due to reproduction, poisoning, and cardiovascular diseases with extreme concentration in PM_2.5_ & PM_10_ over lags 0-24h in Shenzhen from 2013-19

**Figure S4.** Sensitivity analysis: lag structures for the associations of all-cause ambulance emergency calls with extreme concentration in PM_2.5_ & PM_10_ over lags 0-24h in Shenzhen from 2013-19

**Figure S5.** Sensitivity analysis: lag structures for the associations of ambulance emergency calls due to cardiovascular, respiratory, and reproductive diseases with each interquartile range increase concentration in PM_2.5_ & PM_10_ over lags 0-24h in Shenzhen from 2013-19

**Figure S6.** Sensitivity analysis: cumulative concentration-response curves for the association of all-cause ambulance emergency calls with PM_2.5_ & PM_10_ over lags 0-24h in Shenzhen from 2013-19

**Figure S7.** Sensitivity analysis: cumulative concentration-response curves for the associations of ambulance emergency calls due to cardiovascular, respiratory, and reproductive diseases PM_2.5_ & PM_10_ over lags 0-24h in Shenzhen from 2013-19

**Table S1. Distributions of hourly PM_2.5_ & PM_10_ over lags 0-24h prior to the index hour in Shenzhen from 2013-19**

| Air pollutants | Mean | SD | Percentiles | | | | |
| --- | --- | --- | --- | --- | --- | --- | --- |
|  | | | P_2.5_ | P_25_ | Median | P_75_ | P_97.5_ |
| PM_10_ | 49.6 | 24.9 | 15.0 | 28.0 | 42.0 | 63.0 | 120.0 |
| PM_2.5_ | 32.2 | 19.6 | 8.0 | 16.0 | 25.0 | 39.0 | 77.0 |

Abbreviations: SD, standard deviation; P_2.5_, the 2.5 percentile; P_25,_ the 50 percentile; P_75_, the 75 percentile; P_97.5_, the 97.5 percentile; PM_10_, particulate matter less than 10 µm in diameter; PM_2.5_, particulate matter less than 2.5 µm in diameter

**Table S2. Spearman correlation coefficients between air pollutants at an hourly level**

|  | PM_2.5_ | PM_10_ |
| --- | --- | --- |
| PM_2.5_ |  | 0.93 |
| PM_10_ | 0.93 |  |
| NO_2_ | 0.61 | 0.64 |
| O_3_ | 0.56 | 0.52 |
| SO_2_ | 0.62 | 0.66 |

Abbreviations: SD, standard deviation; P_2.5_, the 2.5 percentile; P_25,_ the 50 percentile; P_75_, the 75 percentile; P_97.5_, the 97.5 percentile; PM_10_, particulate matter less than 10 µm in diameter; PM_2.5_, particulate matter less than 2.5 µm in diameter; NO_2_, nitrogen dioxide; O_3_, ozone; SO_2_, sulfur dioxide

Note: All pairwise correlation coefficients were statistically significant (p-value <.05)

**Table S3. Risk of ambulance emergency calls associated with extreme ^a^ concentration in PM_2.5_ & PM_10_ over different lags in Shenzhen from 2013-19**

| AECs | 0-12h | | 0-24h | | 0-36h | | 0-48h | |
| --- | --- | --- | --- | --- | --- | --- | --- | --- |
|  | PM_2.5_ | PM_10_ | PM_2.5_ | PM_10_ | PM_2.5_ | PM_10_ | PM_2.5_ | PM_10_ |
| All-cause | 5.8 (3.0, 8.7) | 6.4 (3.4, 9.5) | 6.3 (2.5, 8.2) | 6.9 (3.8, 10.0) | 5.8 (1.6, 8.1) | 6.8 (3.4, 10.4) | 4.4 (1.5, 7.3) | 6.1 (3.0, 9.4) |
| Cardiovascular | 5.9 (1.1, 10.9) | 6.5 (1.4, 11.9) | 6.5 (0.8, 10.5) | 7.2 (2.0, 12.7) | 5.8 (-0.6, 10.5) | 7.1 (1.2, 13.3) | 4.7 (-0.2, 9.8) | 6.6 (1.2, 12.2) |
| Respiratory | 6.5 (-4.6, 18.9) | 8.5 (-2.7,21.0) | 8.4 (-3.0, 21.2) | 10.1 (-1.4,22.8) | 8.2 (-4.2, 22.2) | 8.2 (-3.9,21.9) | 6.2 (-4.8, 20.6) | 4.7 (-6.5,17.2) |
| Reproduction | 7.7 (-1.9, 18.2) | 7.1 (-4.8, 20.3) | 6.1 (-3.2, 16.3) | 7.7 (-1.5, 17.8) | 4.6 (-5.2, 15.5) | 7.8 (-2.2,14.9) | 8.2 (-1.6, 18.8) | 6.5 (-3.1, 25.3) |

Abbreviations: AECs, ambulance emergency calls; PM_2.5_, PM_10_, particulate matter less than 2.5,10 µm in diameter

Values are estimated percent change (95% CI)

^a^ Extreme: extreme concentration of PM_2.5_ was defined as 97.5^th^ percentile (78.0 µg/m^3^); extreme concentration of PM_10_ was defined as 97.5^th^ percentile (120.0 µg/m^3^)

Models adjusted public holidays, days of the week, hourly temperature, and hourly humidity

**Table S4. Risk of ambulance emergency calls associated with extreme ^a^ concentration in PM_2.5_ & PM_10_ over lags 0-24h stratified by sex, age, season, and time of day in Shenzhen from 2013-19**

| Subgroups | All-cause | | Cardiovascular diseases | | Respiratory diseases | | Reproductive diseases | |
| --- | --- | --- | --- | --- | --- | --- | --- | --- |
|  | PM_2.5_ | PM_10_ | PM_2.5_ | PM_10_ | PM_2.5_ | PM_10_ | PM_2.5_ | PM_10_ |
| Sex | | | | | | | | |
| Male | 5.0 (2.2,8.0) | 6.5 (3.4,9.7) | 5.3 (0.5,10.4) | 6.9 (1.7,12.5) | 7.8 (-3.8,20.7) | 10.0 (-2.8,24.4) | 7.1 (-2.7,17.8) | 8.3 (-2.3,19.9) |
| Female | 5.2 (2.3,8.1) | 6.6 (3.5,9.8) | 5.5 (0.6,10.5) | 7.1 (1.7,12.7) | 7.9 (-3.7,20.9) | 10.1 (-2.7,24.6) | 7.3 (-2.4,17.9) | 8.6 (-2.0,20.3) |
| Age group | | | | | | | | |
| 18-64 | 5.0 (2.2,7.9) | 6.5 (3.3,9.7) | 5.3 (0.5,10.4) | 6.9 (1.6,12.5) | 7.7 (-3.9,20.7) | 9.9 (-2.8,24.8) | 7.0 (-2.6,17.5) | 8.2 (-2.3,19.9) |
| ≥65 | 5.3 (2.1,8.6) | 7.3 (3.8,11.0) | 5.8 (0.5,11.3) | 7.8 (2.0,13.9) | 7.9 (-4.8,22.3) | 10.0 (-3.9,25.8) | 6.7 (-5.1,19.9) | 8.9 (-4.0,23.5) |
| Season ^b^ | | | | | | | | |
| Warm | 4.7 (1.1,8.5) | 8.1 (4.0,12.5) | 1.9 (-4.1,8.4) | 5.1 (-1.9,12.5) | 8.9 (-7.7,28.5) | 12.6 (-4.3,32.6) | 12.6 (-12.8,45.4) | 9.6 (-3.5,24.6) |
| Cool | 7.7 (-31.8,9.5) | -6.8 (-17.2,5.0) | 6.1 (-0.8,13.4) | -12.1 (-28.1,7.4) | 6.5 (-10.2,26.3) | 4.8 (-12.1,24.9) | 13.1 (-18.4,56.9) | 2.5 (-12.1,19.4) |
| Time of Day ^c^ | | | | | | | | |
| Daytime | 5.8 (1.6,10.1) | 7.7 (3.1,12.5) | 5.6 (-0.5,12.0) | 7.3 (0.7,14.3) | 8.5 (-6.5,26.0) | 11.6 (-5.1,31.1) | 5.4 (-10.3,23.9) | 8.1 (-9.3,28.7) |
| Nighttime | 4.9 (1.1,8.8) | 6.2 (2.1,10.4) | 6.2 (-2.2,13.3) | 7.8 (-1.3,15.6) | 5.7 (-9.5,30.7) | 9.2 (-9.1,31.3) | 6.4 (-5.0,19.3) | 7.1 (-5.3,21.1) |

Abbreviations: PM_2.5_, PM_10_, particulate matter less than 2.5,10 µm in diameter

Values are estimated percent change (95% CI)

^a^ Extreme: extreme concentration of PM_2.5_ was defined as 97.5^th^ percentile (78.0 µg/m^3^); extreme concentration of PM_10_ was defined as 97.5^th^ percentile (120.0 µg/m^3^)

^b^ Season: warm is from 1^st^ April to 31^st^ October of each year; cool is from 1^st^ November to 31^st^ March of each year

^c^ Time of the day: Daytime is from 08:00 to 19:00 during one day; Nighttime is from 20:00 to 07:00 the next day

Models adjusted public holidays, days of the week, hourly temperature, and hourly humidity


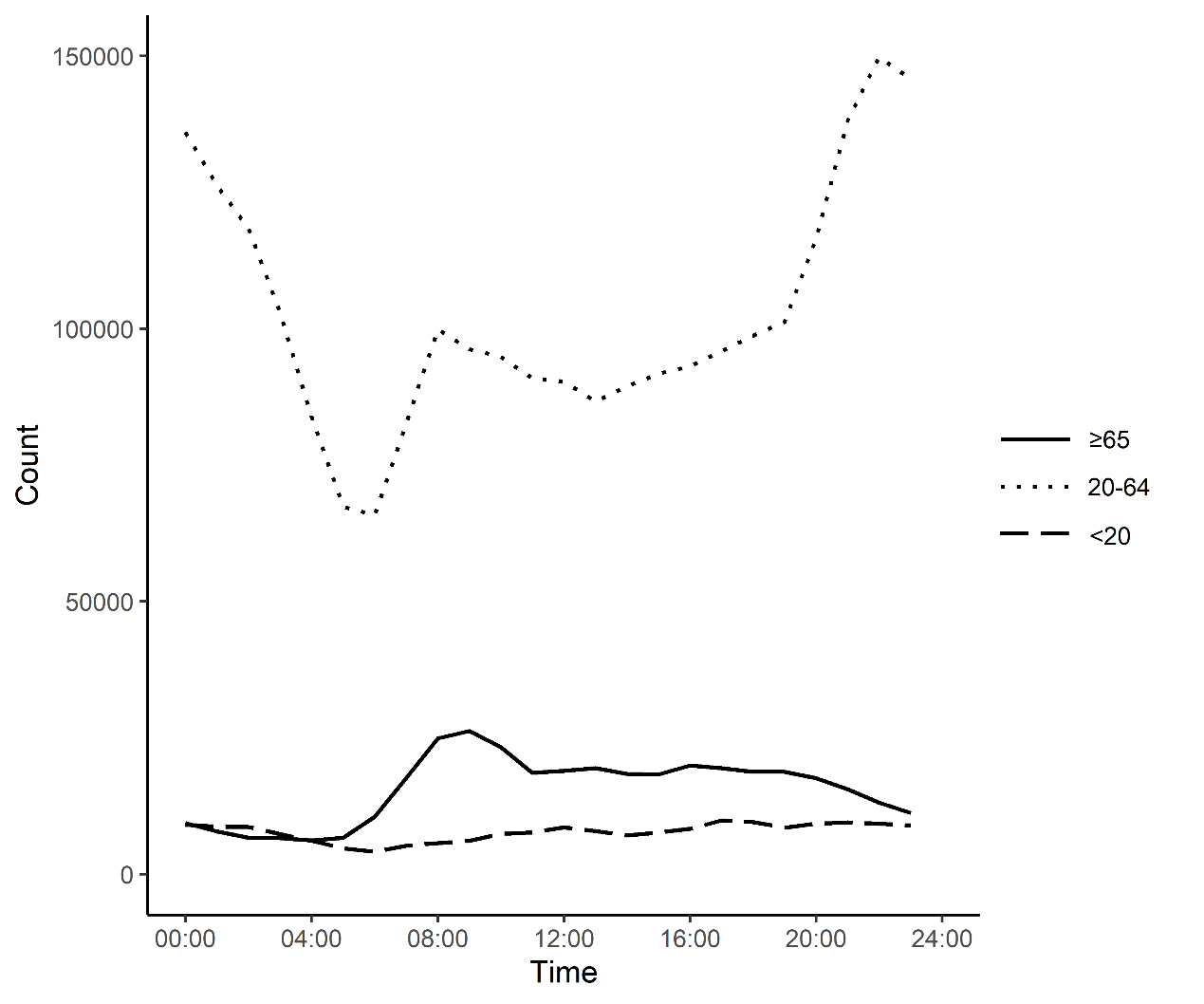


**Figure S1. All-cause ambulance emergency calls’ distribution of 3,022,164 patients over 0-24h, stratified by age in Shenzhen from 2013-19**


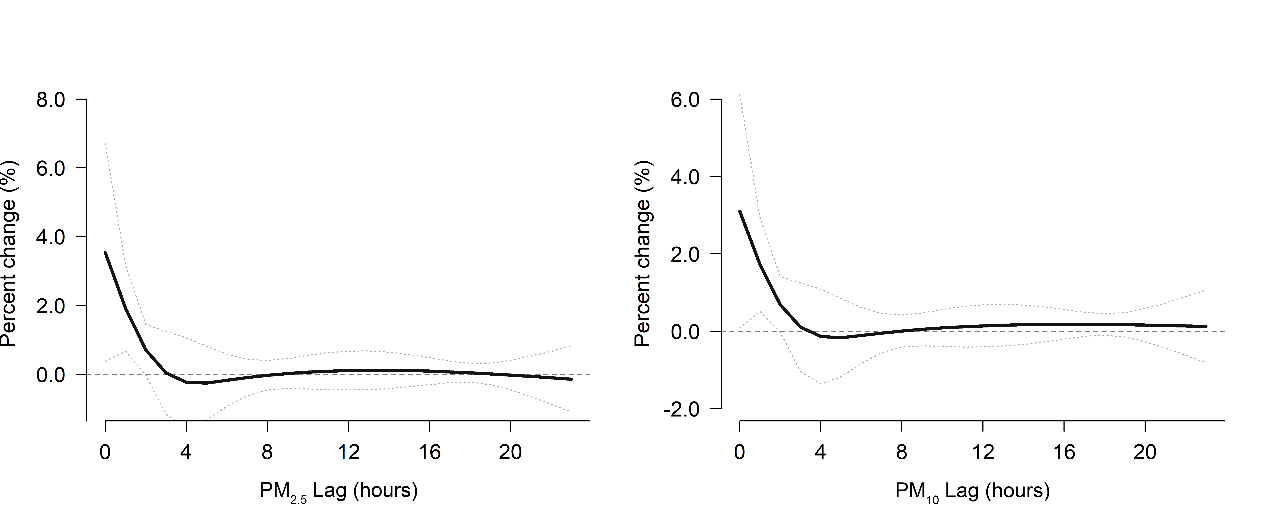


**Figure S2. Lag structures for the associations of all-cause ambulance emergency calls with extreme concentration in PM_2.5_ & PM_10_ over lags 0-24h in Shenzhen from 2013-19.**

The overall lag structure curves are calculated using a linear with two knots placed on the log scale of lags to model the lag-response association. The black solid lines are the average percentage change in the risk of all-cause ambulance emergency calls with extreme concentrations of PM_2.5_ & PM_10_ (78.0 µg/m^3^, 120.0 µg/m^3^), and the dotted lines are the 95% confidence intervals. Abbreviations: PM_2.5_, PM_10_, particulate matter less than 2.5,10 µm in diameter.


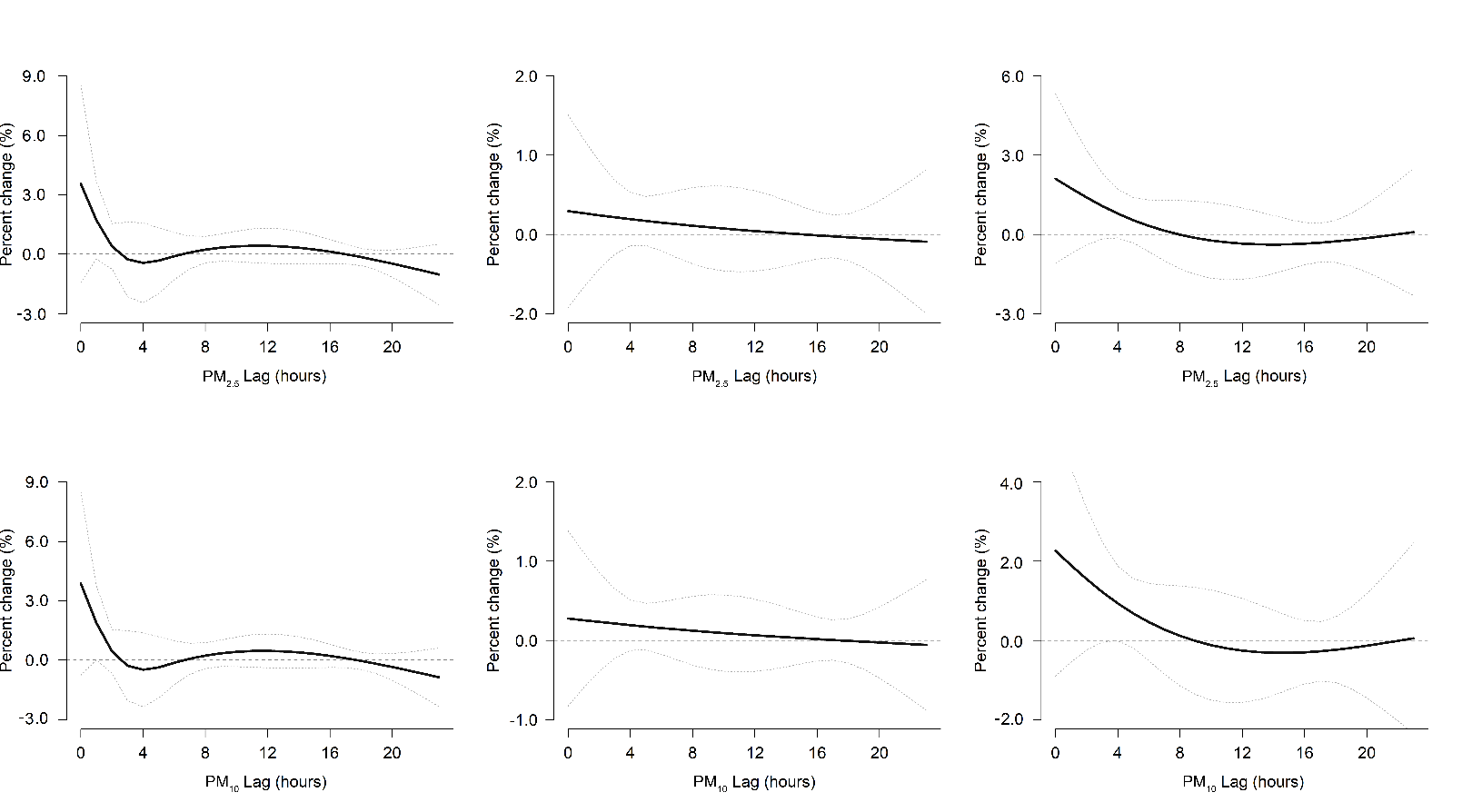


**Figure S3. Lag structures for the associations of ambulance emergency calls due to reproduction, poisoning, and cardiovascular diseases with extreme concentration in PM_2.5_ & PM_10_ over lags 0-24h in Shenzhen from 2013-19**

A & D represent cardiovascular diseases, B & E represent respiratory diseases, and C & F represent reproductive illnesses. The overall lag structure curves are calculated using a linear with one or two knots placed on the log scale of lags to model the lag-response association. The black solid lines are the average percentage change in the risk of ambulance emergency calls due to cardiovascular, respiratory, and reproductive diseases with extreme concentrations of PM_2.5_ & PM_10_ (78.0 µg/m^3^, 120.0 µg/m^3^), and the dotted lines are the 95% confidence intervals. Abbreviations: PM_2.5_, PM_10_, particulate matter less than 2.5,10 µm in d


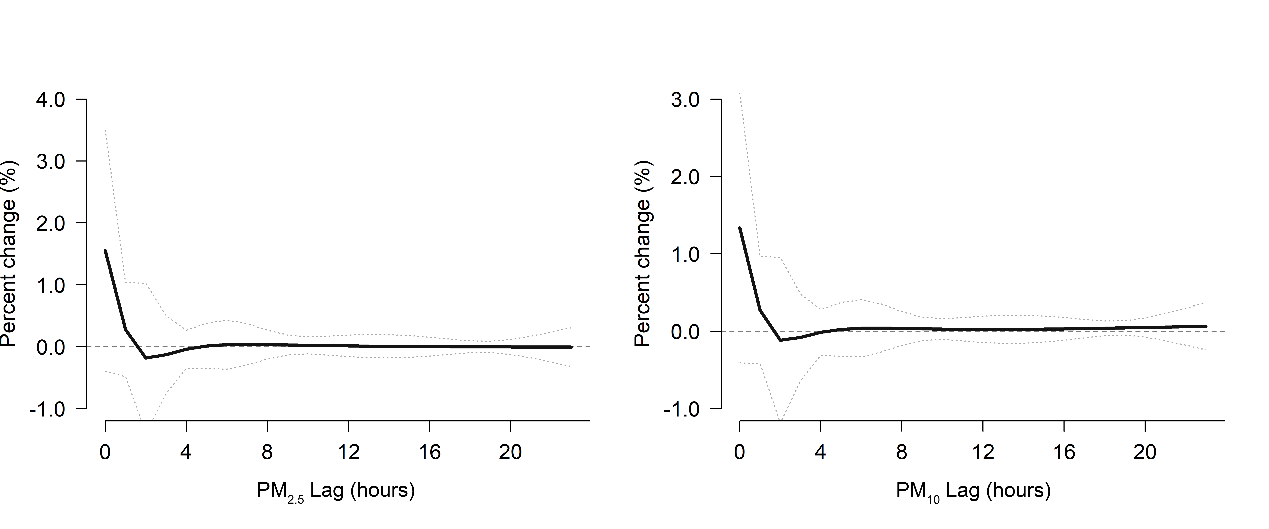


**Figure S4. Sensitivity analysis: lag structures for the associations of all-cause ambulance emergency calls with extreme concentration in PM_2.5_ & PM_10_ over lags 0-24h in Shenzhen from 2013-19**

The overall lag structure curves are calculated using a linear with three knots placed on the log scale of lags to model the lag-response association. The black solid lines are the average percentage change in the risk of all-cause ambulance emergency calls with each IQR increase in PM_2.5_ & PM_10_ (24.0 µg/m^3^, 34.0 µg/m^3^), and the dotted lines are the 95% confidence intervals. Abbreviations: IQR, interquartile range; PM_2.5_, PM_10_, particulate matter less than 2.5,10 µm in diameter.


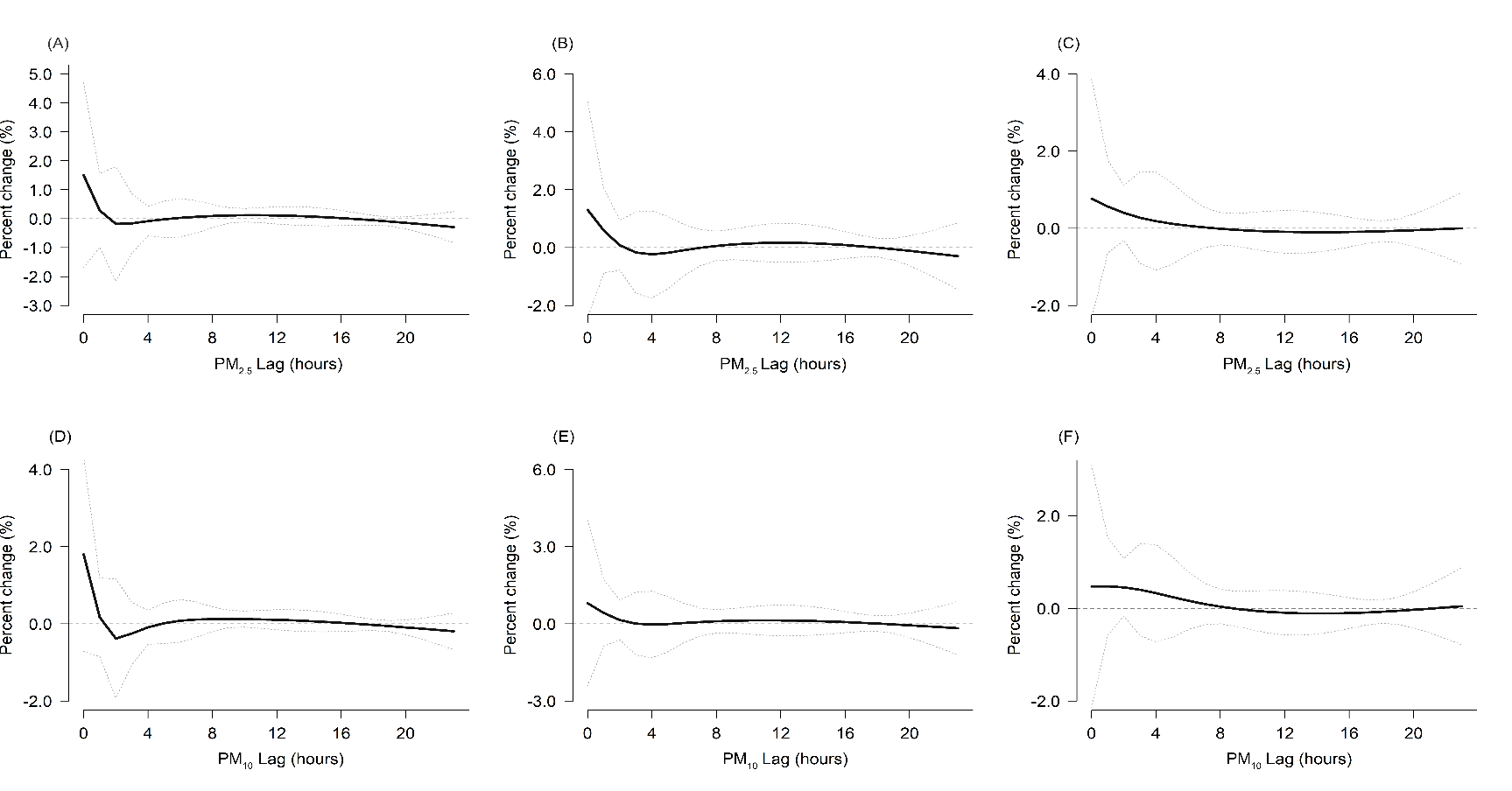


**Figure S5. Sensitivity analysis: lag structures for the associations of ambulance emergency calls due to cardiovascular, respiratory, and reproductive diseases with each interquartile range increase concentration in PM_2.5_ & PM_10_ over lags 0-24h in Shenzhen from 2013-19**

A & D represent cardiovascular diseases, B & E represent respiratory diseases, C & F represent reproductive illnesses. The overall lag structure curves are calculated using a linear with two or three knots placed on the log scale of lags to model the lag-response association. The black solid lines are the average percentage change in the risk of ambulance emergency calls due to cardiovascular, respiratory, and reproductive diseases with extreme concentrations of PM_2.5_ & PM_10_ (24.0 µg/m^3^, 34.0 µg/m^3^), and the dotted lines are the 95% confidence intervals. Abbreviations: IQR, interquartile range; PM_2.5_, PM_10_, particulate matter less than 2.5,10 µm in diameter.


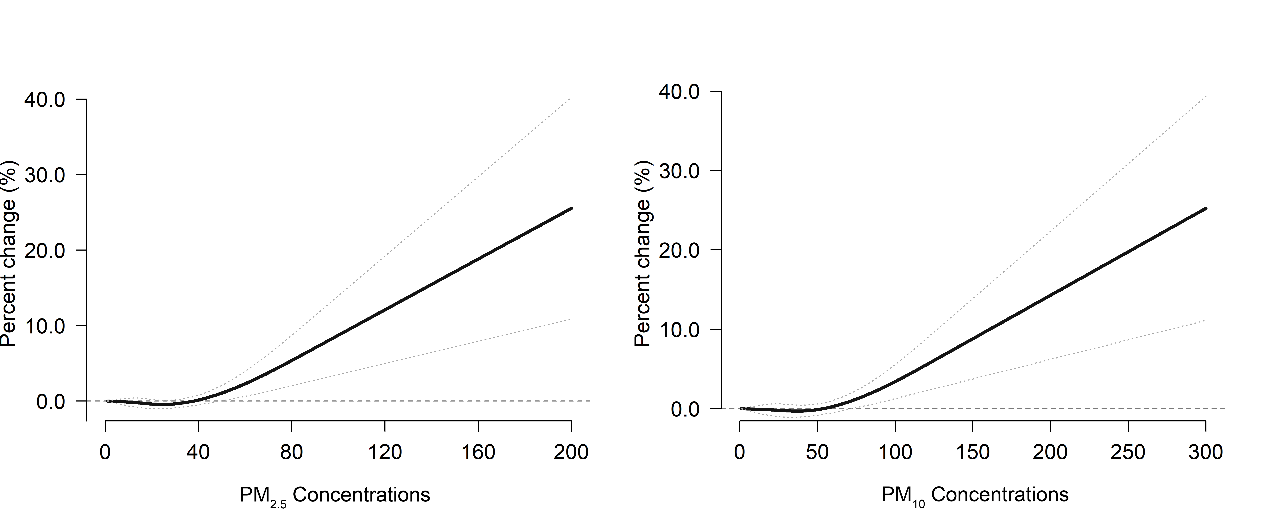


**Figure S6. Sensitivity analysis: cumulative concentration-response curves for the association of all-cause ambulance emergency calls with PM_2.5_ & PM_10_ over lags 0-24h in Shenzhen from 2013-19**

The cumulative exposure-response curves are calculated using a natural B-spline with two knots to model the exposure-response association. The black solid lines are the average percentage change in the risk of all-cause ambulance emergency calls, and the dotted lines are the 95% confidence intervals. Abbreviations: PM_2.5_, PM_10_, particulate matter less than 2.5,10 µm in diameter.


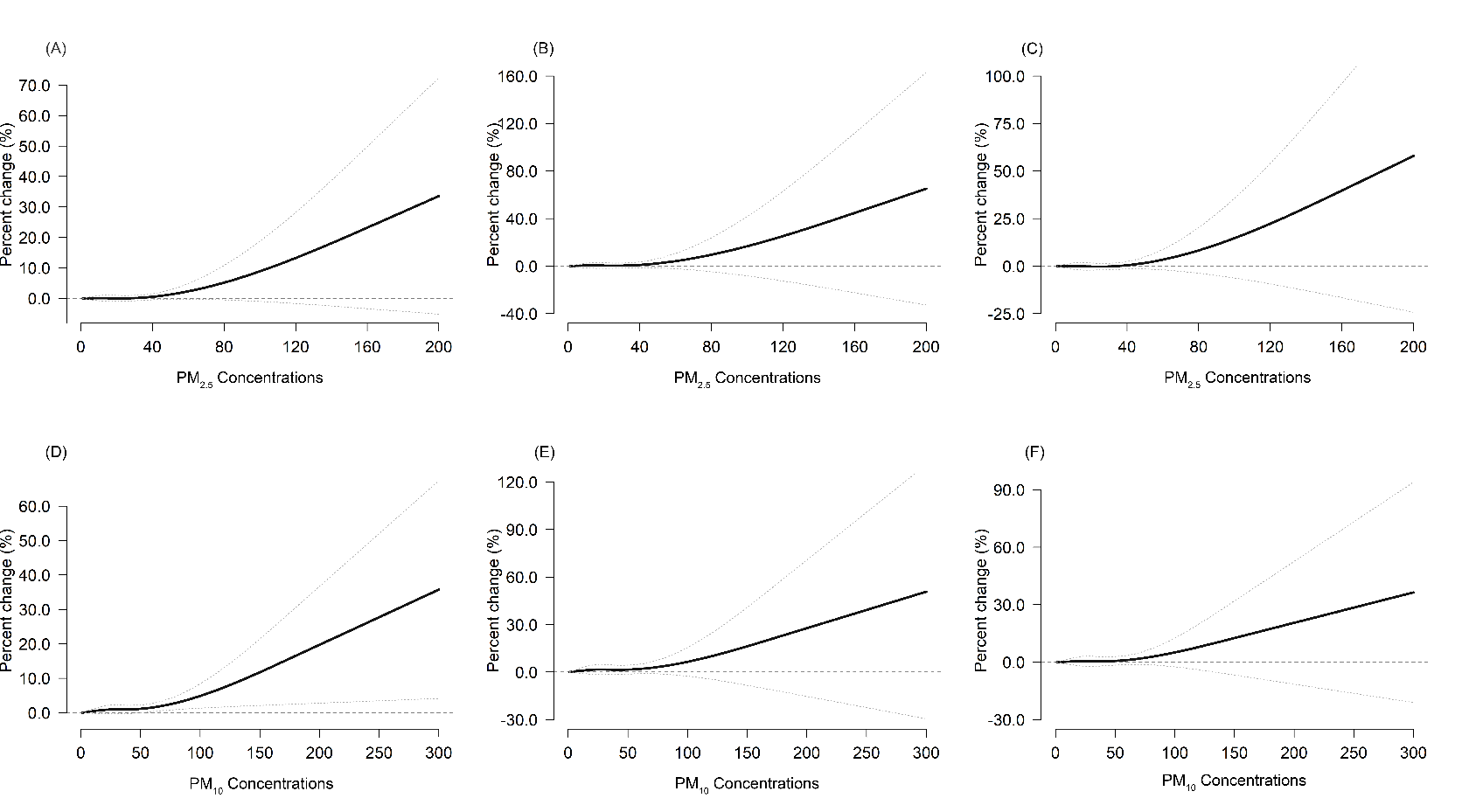


**Figure S7. Sensitivity analysis: cumulative concentration-response curves for the associations of ambulance emergency calls due to cardiovascular, respiratory, and reproductive diseases with PM_2.5_ & PM_10_ over lags 0-24h in Shenzhen from 2013-19**

A & D represent cardiovascular diseases, B & E represent respiratory diseases, C & F represent reproductive illnesses. The cumulative exposure-response curves are calculated using a natural B-spline with two or three knots to model the exposure-response association. The black solid lines are the average percentage change in the risk of ambulance emergency calls due to cardiovascular, respiratory, and reproductive diseases with PM_2.5_ & PM_10_, and the dotted lines are the 95% confidence intervals. Abbreviations: PM_2.5_, PM_10_, particulate matter less than 2.5,10 µm in diameter.
